# The Occurrence of Microplastics in *Donax trunculus* (Mollusca: Bivalvia) Collected along the Tuscany Coast (Mediterranean Sea)

**DOI:** 10.3390/ani14040618

**Published:** 2024-02-14

**Authors:** Chiara Malloggi, Luca Nalbone, Silvia Bartalena, Margherita Guidi, Carlo Corradini, Antonino Foti, Pietro G. Gucciardi, Filippo Giarratana, Francesca Susini, Andrea Armani

**Affiliations:** 1 FishLab, Department of Veterinary Sciences, University of Pisa, Viale delle Piagge 2, 56124 Pisa, Italy; chiaramalloggi01@gmail.com (C.M.); m.guidi21@studenti.unipi.it (M.G.); 2 Department of Veterinary Sciences, University of Messina, Polo Universitario Dell’Annunziata, 98168 Messina, Italy; luca.nalbone@unime.it (L.N.); filippo.giarratana@unime.it (F.G.); 3 Experimental Zooprophylactic Institute of Latium and Tuscany M. Aleandri, UOT Toscana Nord, SS Dell’ Abetone e del Brennero 4, 56123 Pisa, Italy; s.bartalena@gmail.com (S.B.); francesca.susini@izslt.it (F.S.); 4 Experimental Zooprophylactic Institute of Latium and Tuscany M. Aleandri, Via Appia Nuova, 1411, 00178 Roma, Italy; carlo.corradini@izslt.it; 5 Consiglio Nazionale delle Ricerche (CNR), Istituto per i Processi Chimico-Fisici (IPCF), Viale F. Stagno D’Alcontres 27, 98158 Messina, Italy; antonino.foti@cnr.it (A.F.); pietrogiuseppe.gucciardi@cnr.it (P.G.G.)

**Keywords:** microplastics, bivalve molluscs, *Donax trunculus*, risk assessment, consumer protection

## Abstract

**Simple Summary:**

Microplastics (MPs) are anthropogenic microscopic pieces of plastic in marine sediments and the water column that can originate from two different sources. Primary MPs are industrially produced microbeads, while secondary MPs result from the fragmentation of larger plastic debris by physical, chemical, and biological processes. Although not yet considered a food contaminant, they are readily ingested by marine organisms at several trophic levels, thus entering the food chain and representing a risk to consumers of fishery products. In this study, *Donax trunculus* (Truncate donax) specimens collected from Class A production areas in the Tyrrhenian Sea (Mediterranean Sea), and therefore destined directly for the final consumer, were analyzed for the presence of MPs. First, all items were morphologically classified and measured, and some of these were chemically identified. Then, the MPs’ mean abundance (MA) was calculated, and a risk assessment of human exposure to MPs was carried out. Both the MA of MPs and human exposure to MPs were found to be low. However, given the magnitude of the problem, the collection of further data using standardized methods is essential for a better risk assessment.

**Abstract:**

Microplastics (MPs) (0.1 µm–5 mm particles) have been documented in oceans and seas. Bivalve molluscs (BMs) can accumulate MPs and transfer to humans through the food chain. BMs (especially mussels) are used to assess MPs’ contamination, but the genus *Donax* has not been thoroughly investigated. The aim of this study was to detect and characterize MPs in *D. trunculus* specimens collected along the Tuscan coast (Italy), and to assess the potential risk for consumers. The samples (~10 g of tissue and intervalval liquid from 35 specimens) were digested using a solution of 10% KOH, subjected to NaCl density separation, and filtered through 5 μm pore-size filters. All items were morphologically classified and measured, and their mean abundance (MA) was calculated. Furthermore, 20% of them were analyzed by Raman spectroscopy and, based on the obtained results, the MA was recalculated (corrected MA) and the annual human exposure was estimated. In the 39 samples analyzed, 85 items fibers (*n* = 45; 52.94%) and fragments (n = 40; 47.06%) were found. The MA was 0.23 ± 0.17 items/grww. Additionally, 83.33% of the items were confirmed as MPs (polyethylene and polyethylene terephthalate). Based on the correct MA (0.18 MPs/grww), *D. trunculus* consumers could be exposed to 19.2 MPs/per capita/year. The health risk level of MPs was classified as level III (moderate).

## 1. Introduction

The term microplastics (MPs) is used to describe a mixture of differently shaped plastic materials in the range of 0.1 µm–5 mm in marine sediments and in the water column [1,2]. MPs could have two different origins. Primary MPs are industrially manufactured microbeads (e.g., abrasive particles, powders for injection molding, resin pellets for bulk transportation of polymers between manufacturing sites), while secondary MPs derive from the fragmentation of larger plastic debris during their use (e.g., textile and rope fibers, weathering and the fragmentation of larger litter items, vehicle tire wear, paint flakes) or as a consequence of weathering degradation processes, especially in the marine environment [3,4]. MPs are therefore abundant in both the water column and in sediments. Depending on their density, MPs can float on the surface of the water (low density) or sink (high density) and accumulate [5,6]. Moreover, as degradation and physical breakdown proceeds, their total number magnifies [7]. MPs can also accumulate in different aquatic organisms by entering through multiple pathways, such as filter feeding, suspension feeding, direct ingestion, and trophic transfer through the consumption of exposed preys [8,9,10]. In this way, they can be transferred through food webs [11,12]. Thus, MPs should be considered as a food contaminant with a potential health threat for seafood consumers [8,13,14]. Despite the risk of exposure to MPs for human health being largely unknown, it is recognized that they can release additives and toxins that, once absorbed, can cause both physical and chemical stress to the human digestive system [15,16]. Further research to properly assess the human risks linked to MPs is therefore required [17,18].

Bivalve molluscs (BMs) are an important source of human food, and they are characterized by a high commercial interest worldwide [19]. Because of their filter-feeding activity [20], BMs are able both to accumulate and concentrate different kinds of contaminants [21,22]. Regarding MPs, part of them can be not egested by BMs, and they consequently accumulate in different organs based on their chemical and physical characteristics [23]. Therefore, considering that BMs are eaten whole, BMs can expose consumers to this kind of potential hazard [24].

Among BMs, mussels are the most analyzed for MPs detection, followed by oysters, clams, and scallops [25]. BMs of the Genus Donax (Ansell, 1893) (superfamily Tellinoidea, Family Donacidae) are distributed along sandy coastlines in tropical and temperate zones, including the Mediterranean Sea [26]. *D. trunculus* (Truncate donax) represents an important resource in France, Italy, Portugal, Spain, and Turkey [27]. This species is a constituent of the infra-littoral benthic fauna of well-graded fine sands, and it especially inhabits the wash zones of the beaches (at depths between 0 and about 2 m) where it feeds mainly off phytoplankton and organic material [28,29,30]. Therefore, MPs can be assimilated by *D. trunculus*, firstly, by gills, and then by the digestive gland where they seem to be accumulated [10,31]. While *D. trunculus* has been extensively used to monitor other types of marine pollution in Mediterranean areas [10], only five studies assessed the occurrence of MPs in this species (Table 1). Only three of them carried out polymer identification (see Table 2 for details). Of these, only two [32,33] analyzed specimens from the Mediterranean Sea finding fibers and fragments composed by different polymers (see Table 2 for details). Interesting to note, these two studies are rather recent, the first of them, by Olivieri et al. [33], being published in 2020. This study [33] was also the only one from the Tyrrhenian Sea along the Italian cost (Latium Region) (Table 1). In this study, the need to conduct further research to confirm the accumulation of specific MP shapes in *D. trunculus* from Mediterranean Sea was emphasized [33].

The Mediterranean Sea, with the highest concentrations of floating plastic particles worldwide, is one of the most impacted areas in terms of MPs [37,38]. The coasts of Latium and Tuscany (Tyrrhenian Sea) represent about 1000 of the 8000 km of the Italian coastline. Currently, the collection of *D. trunculus* along the Tuscany coast is allowed from natural banks located in sea areas, classified as Class A areas by the Local Health Authority (LHA) [39,40]. *D. trunculus* from these areas are therefore intended for direct human consumption without any treatment in a depuration center [41]. The aim of this study was to investigate the occurrence of MPs in *D. trunculus* specimens from the Mediterranean Sea (Tyrrhenian Sea) along the coasts of the Tuscany region and characterize them in terms of polymer composition. In addition, the potential risk for consumers was assessed. This study can therefore concur to better describe the MPs occurrence in poor areas (Tuscany coast).

## 2. Materials and Methods

### 2.1. Specimens’ Collection

**Figure 1:**
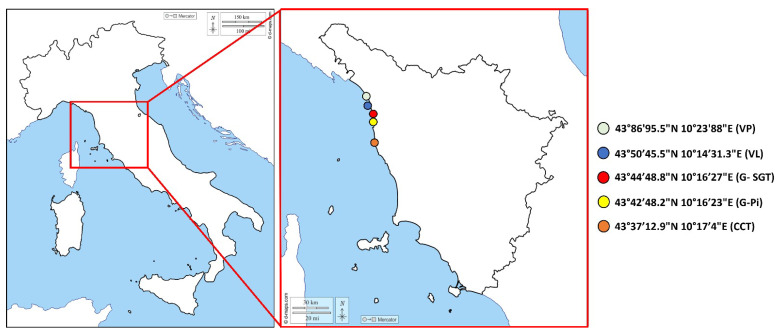
Areas of *D. trunculus* harvesting along the Tuscany coast, and relative collection points with coordinates: Viareggio Ponente (VP), Viareggio Levante (VL), Gombo San Giuliano Terme (G-SGT), Gombo Pisa (G-Pi), and Centro Coni and Tirrenia (CCT).

According to the Community Guide to the Principles of Good Practice for the Microbiological Classification and Monitoring of Bivalve Molluscs Production and Relaying Areas, with regard to Implementing Regulation 2019/627 [42], LHA have to designate collection points in order to ensure that the results obtained from samples are representative of the degree of contamination of the entire area. In addition, a representative BMs sampling program in term of the number of samples, geographical distribution and frequency sampling (Commission Implementing Regulation (EU) No 2019/627, Article 56) must be defined [40]. Accordingly, *D. trunculus* specimens were collected in 2021 by the LHA of the Tuscany region from five collection points located within five production areas (Class A) (Viareggio Ponente (VP), Viareggio Levante (VL), Gombo San Giuliano Terme (G-SGT), Gombo Pisa (G-Pi), and Centro Coni and Tirrenia (CCT), as defined by the Guidelines provided by the Tuscany Regional Council Resolution 1401 of 11 December 2017 [43] (Figure 1). No collections were performed in January, due to the bad weather conditions, or in April, during which the harvesting of *D. trunculus* is prohibited for restocking purposes (Ministerial Decree of 22 December 2000 of the Ministry of Agriculture and Forestry (Fisheries and Aquaculture)) [44].

The collection was performed from February to December 2021. All the collected specimens were stored in aluminum foil to avoid any types of contamination, and then transported at freezing temperature (−20 °C) to the Experimental Zooprophylactic Institute of Latium and Tuscany (IZSLT) (Pisa section). Each collection, composed of 50 specimens, was associated to an internal code (Table S1). Some specimens from the same collection points, but collected in 2020, were used as positive control (see Section 2.6).

### 2.2. Sample Preparation and Items Isolation (Digestion and Filtration)

All the collected specimens belonging to the samples collected in this study (Table S1) were defrosted and shelled using a metal scalpel to constitute a sample of ~10 g (accuracy of 0.001 mg) of tissue and intervalval liquid of 35 specimens. All the samples were processed using the protocol proposed by Ben-Haddad et al. [32] for the detection of MPs in *D. trunculus,* based on digestion with 10% KOH (Sigma-Aldrich, St. Louis, USA) solution, followed by a flotation with NaCl. The protocol was slightly modified with respect to the digestion time in 10% KOH which was reduced from the 72 h reported in the original protocol to 48 h by inserting an oscillator directly into the oven. Indeed, from the preliminary tests, it was observed that the digestion effectiveness was not influenced by decreasing the digestion times. Once digested, samples were removed from the oven and added with a concentrated saline solution in double-filtered deionized water (1.2 gr/mL NaCl > 99%; Sigma-Aldrich) to separate items from sediments, fecal casts, and *D. trunculus* matter. Furthermore, 250 mL of the NaCl solution was added to each sample (1:1 *v*/*v*), which was again covered with aluminum foil and transferred under a fume hood at room temperature overnight (ON). After the ON density separation, 200 mL of supernatant was collected using a 25 mL glass pipette through concentric movements from the walls to the center of the beaker. The supernatant was filtered using a device (BioSigma, Milan, Italy) connected to a vacuum pump through a cellulose-mixed ester membrane filters (Ø 47 mm; Whatman with 5 μm pore size). Each filter was then placed in a covered 90 mm Ø glass Petri dish (Biosigma)) and left to dry for a few hours, waiting for stereomicroscope observation. Overall, seven sessions (comprising five or six samples each) of digestion and filtering were performed.

### 2.3. Filter Observation

The filters obtained from each sample were observed to detect potentially plastic items differently from organic materials and sand [45,46]. Each filter was observed three times by two operators (under the same conditions) to confirm the number of detected items. Only items < 5 mm in size with a uniform color and thickness (in the case of fibers) were considered during the observation [47]. Moreover, items were checked to assess their nature (plastics or organic). If items disintegrate when touched with a needle, they were considered organic residues ‘surviving’ digestion [48]. The items observed in the filters of the samples were compared (in terms of size, color, and shape) with those detected on the filters of the procedural blanks and blank filters of the same analysis session (see Section 2.6). Items resemblant to those observed in blanks were excluded from the count. The retained items were subsequently classified morphologically as proposed by the Group of Experts on the Scientific Aspects of Marine Environmental Protection for MPs [14,47].

To measure items, images obtained by observing filters at a 40X magnification were captured with the aid of a camera (Apple iPhone 11; 12 MPX ƒ/1.8). Subsequently, the major axis of each photographed item was measured using ImageJ software (version 1.52t; https://imagej.nih.gov/ij/download.html, accessed on 25 November 2023). In addition, the items were also classified according to their length using the following intervals: (a) 25–50 µm; (b) 50–100 µm; (c)101–200 µm; (d) 201–500 µm; (e) 501–1000 µm; (f) 1001–1500 µm; (g) 1501–2000 µm; (h) 2001–3000 µm; (i) >3001 µm [47]. The morphology, color, and size of each item were recorded.

### 2.4. Items Abundance and Statistical Analysis

First, the item abundance per sample (pool = 10 g) was calculated as follows:

$$Items=\frac{Total\;number\;of\;items\;in\;the\;sample\;(pool)}{Total\;wet\;weight\;of\;the\;sample\;(pool)(10gr)}$$

Then, the overall mean abundance (MA) (mean ± standard deviation) of the item was calculated. Moreover, each sample was assigned to categories based on the number of items found: (a) no items (0); (b) 1 or 2 items; (c) >2 items. Finally, the percentage of positive samples (samples in which at least one item was found) per site and per season was calculated.

### 2.5. Polymer Identification

#### 2.5.1. Chemical Identification of the Items

**Figure 2:**
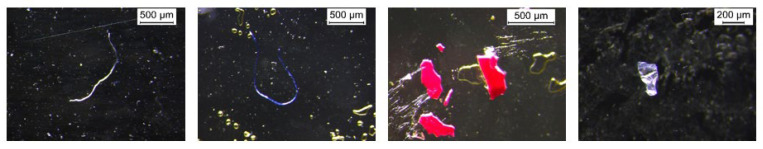
Items extracted from samples of *D. trunculus,* analyzed for MP detection. The items were observed under a stereomicroscope and selected for the chemical identification of their constituent polymers.

Approximately 20% of the items found were analyzed to determine their chemical composition, identifying the constituent polymers by Raman spectroscopy. The items to be tested were randomly selected among those extracted from the samples, based on their morphological prevalence. In detail, twelve MPs’ fibers (ranging in size between ~150 μm and ~1500 μm) and six MPs’ fragments (ranging in size between ~50 μm and ~1000 μm) were analyzed. Figure 2 shows some of the items analyzed.

Items were transferred from the filters’ surface onto scotch tape previously attached to glass slides, and subsequently covered with a glass coverslip to prevent further contamination. Each item was first spotted under the stereomicroscope, and then the correspondent area was analyzed with the microspectrometer for the Raman characterization.

#### 2.5.2. Raman Analysis

Raman characterizations were carried out using an XploRA Plus microspectrometer (Horiba Scientific, Kyoto, Japan), equipped with a diode laser emitting at 785 nm. The acquisition time was set at 20 s, and the laser power was set at 37 mV. The measurements were performed at room temperature using either a 100× or 50× long working distance microscope objective (Evident corporation-Olympus, Tokyo, Japan),which helped focus the laser beam onto the sample surface. The scattered light was collected in a backscattered configuration through the same objective, and subsequently was dispersed by a diffraction grating (1200 lines/mm) onto a CCD detector (Syncerity, Horiba Scientific, Rome, Italy). The vibrational fingerprint of each item was compared with a database of reference polymer spectra using commercial software (SpectraGenius v1.3.p—S.T. Japan-Europe GmbH, Cologne, Germany). Only a match of at least 70% was considered reliable [49,50,51]. The Raman spectrum of each item was analyzed after the subtraction of the glass contribution and the removal of the fluorescence background (see Figure S1). The Raman spectrum of the scotch tape was also acquired to avoid any misleading identifications of the items.

### 2.6. Quality Control Measures

To avoid accidental contamination with MPs of environmental origin, the analysis was performed in a restricted access room. All procedures, except for the sample preparation (see Section 2.2) and filter observation, were carried out under a clear airflow cabinet, wearing nitrile gloves and white 100% cotton gowns [48,52]. All materials, equipment, and laboratory surfaces were washed and rinsed with double-filtered deionized water, obtained using sterile syringe filters (cellulose acetate) with 0.22 μm pore size (Ø 25 mm; Millipore, Sigma-Aldrich). All the solutions used in this study were produced using the same water. Only glassware was used. The samples were covered with aluminum foil when at risk of being exposed to airborne particles, and all the experiments were completed as quickly as possible.

One procedural blank (sample without tissue prepared as described in Section 2.3) was included during each session of digestion and filtering. In addition, blank filters, consisting of filters laid on open Petri dishes and placed next to the sample or to the operator during procedures conducted outside the cabinet, were used (one for each observation session). The blank filters were subsequently observed under a stereomicroscope (Olympus SZX9) at a 40× magnification to detect the occurrence of items to monitor environmental contamination during sample processing.

Three positive controls were performed to evaluate both the recovery rate of the method, and to determine the minimum observable particle size with the used stereomicroscope. They were prepared by spiking 20 MPs made of blue polystyrene (bPS) (1.050 g/cm^3^) of a known size range (106–500 µm) into 10 g of *D. trunculus* samples (see Section 2.3), collected in 2020. MPs were obtained by fragmenting bPS plastic plates with an ultra-centrifugal mill (ZM 200, Retsch GmbH, Haan, Germany), equipped with a 500 μm steel mesh sieve. The obtained particles were further sieved with a 106 μm steel mesh column sieve obtaining a batch of MPs ranging in the known size between 106 μm and 500 μm. The positive controls were processed using the protocol described in Section 2.2, and the MPs recovery rate was calculated by counting the number of retrieved particles on the filters (as described in Section 2.6). The minimum detectable particle size was established by spiking white low-density polyethylene (LDPE) (0.924 g/cm^3^; Sigma-Aldrich, USA) MPs of different size ranges (40–48 μm, 63–90 μm; 91–125 μm; 126–180 μm; 181–355 μm; and 356–510 μm) onto white filters, which were then observed under the stereomicroscope at a 40× magnification. In detail, particles were tested in decreasing order of size by spiking 20 particles of each size batch onto a white filter. The batch size of the smallest particles that were easily observable on the filter surface under the stereomicroscope was the minimum detectable particle size of our method.

### 2.7. MP Abundance and Annual Human Exposure

Once the true nature of the items was verified, the MA was calculated again based on the percentage of items identified as MPs. For literature data comparison, considering the extremely high number of studies investigating MPs abundance in BMs, we exclusively considered the data provided by two recent systematic reviews on this topic [25,53]. In addition, the five studies investigating *D. trunculus* (Table 1), or other co-generic species, were considered in the comparison. The MPs consumers’ annual exposure was then calculated. In this case, since no data on the per capita consumption of *D. trunculus* are available, we considered data provided for clams by EUMOFA (2022) [54], corresponding to 320 g of the total body weight (including shell). Since the soft tissue (edible part) of BMs is reported to be almost one-third of the whole-body weight [49], we calculated the annual human exposure to MPs as corresponding to 106.6 g (320/3).

## 3. Results and Discussion

### 3.1. Specimens Collection

Sampling parameters, including collection point and collection date, are important factors in the characterization of MPs contamination, and they should therefore be reported. In addition, considering that the MP concentration is highly dependent on the area, human presence, and the season, with the highest near-heavily populated urban areas [55], detailed sampling information are essential for data interpretation and comparison. However, such information is not always provided in studies published to date [25]. The samples investigated in this study were collected from collection points identified by the LHA of the Tuscany region, as representative of the pollution of the five production areas in which BMs are harvested (see Section 2.1). In particular, the specimens analyzed in this study were collected in the context of official monitoring activities. To the best of our knowledge, no study conducted in the EU for the detection of MPs in BMs mentions the class of the production or relaying area, from which the BMs were collected. In the available studies on MPs in *D. trunculus* (Table 1), the specimens were collected from collection points (from 1 up to 9) selected by researchers, based on the harvesting and marketing areas of this species.

**Figure 3:**
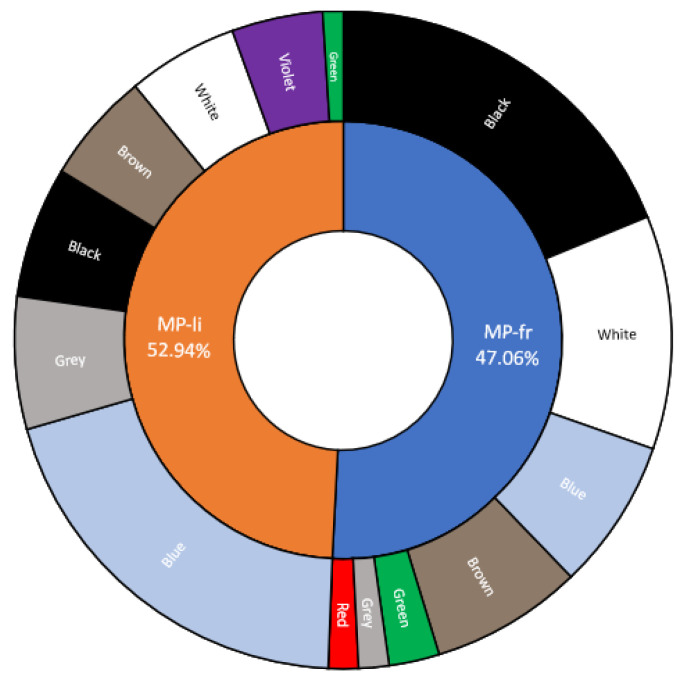
Sunburst diagram showing the shape (fibers (MP-li); fragments (MP-fr)) and color of the extracted items and their relative percentages.

Regarding the sample size, at least 50 specimens per research unit is recommended to represent the population, according to the Marine Strategy Framework Directive [56]. Indeed, more than 50 BM specimens were collected in most of the studies published between 2011 and 2020 [25]. Accordingly, in the five studies conducted on *D. trunculus,* the number of collected specimens (reported only in four) ranged from 51 to 1632 (Table 1). However, it should be considered that the number of collected specimens does not always correspond to those analyzed (Table 1). Also, in this study, 50 specimens of *D. trunculus* per 39 collection date (1950 specimens) were collected and ~1365 specimens were analyzed. This, therefore, represents the second study in terms of the number of *D. trunculus* specimens analyzed for MP detection (Table 1). In detail, nine samplings were performed at G-Pi (23.08%) and G-GT (23.08%), eight (20.51%) at CCT, seven (17.95%) at VL, and the last six at VP (15.38%) (Figure 3). The collection was also distributed along seasons with eighteen (46.15%; *n* = 900) samplings carried out during summer, eight (20.51%; *n* = 400) in spring, seven (17.95%; *n* = 350) in autumn, and six (15.38%; *n* = 300) in winter. The highest number of samples were collected in June (*n* = 9; 450 specimens), followed by March (*n* = 6; 300 specimens), and July/August (*n* = 5; 250 specimens). Literature studies analyzing *D. trunculus* (Table 1) did not cover all the season periods (Table 1). Accordingly, as reported in the review by Ding et al. [25], only in six of the sixty-one analyzed studies regarding the detection of MPs in BMs was the sampling performed in more than one season.

### 3.2. Quality Control Measure

Quality control measures should always be conducted and described for the assessment of the reliability of the results [22]. In this study, strict measures to avoid contamination, which could lead to an overestimation of the number of MPs in the sample, were implemented, starting from the specimen collection. In fact, given their structural heterogeneity, MPs can also be traced in the atmosphere, as well as in the equipment, reagents, and materials used for the extraction process itself, such as gloves, gowns, and solvents [57]. Background contamination from the surrounding environment was avoided throughout sample treatments and analysis. As a result, the procedural blanks only contained 0.29 ± 0.49 items per filter. Seven procedural blanks (one for each digestion session) were produced and analyzed together with the samples to monitor secondary contamination (environment and air) [25,58]. Procedural blanks are effectively widely used as quality control in studies aimed at detecting MPs in BMs. According to the review by Ding et al. [25], 93.4% of the studies on this topic included this kind of quality control. In contrast, only 28% included positive controls to determine the recovery rate [25]. Positive controls were instead included in this study. Among the different size classes tested, the 40–48 μm range was the smallest in which potential LDPE items could be clearly identified under the 40x stereomicroscope magnification. An MP recovery rate of 95% (57 particles found on a total of 60) was defined, allowing an evaluation of the results obtained with a good level of confidence. Indeed, recovery rates between 80 and 100% allow for the obtaining of comparable results [25,48]. In addition, the high recovery rate confirmed the low degradation impact on the MPs of the protocol digestion, in line with the literature [59].

### 3.3. Items Detected in D. trunculus

#### 3.3.1. Items Isolation Protocol

Considering that treatments (like depuration) might affect the true estimation of MPs in the samples, the specimens were subjected to MPs extraction without preliminary washing steps [53]. For the MPs extraction, the protocol of Ben-Haddad et al. [32] was chosen as a starting point, because it was designed for the detection of MPs in *D. trunculus,* and it also used a 10% KOH solution (at 40 °C), which is considered to provide the most efficient removal of soft tissue, while protecting MPs [59]. In fact, digestion with 10% KOH is the most suitable technique for digesting BMs tissues, even when the target particles are only one micrometer in size. In addition, it is a cheaper and less time-consuming method [59]. In the review of Ding et al. [25], 10% KOH is reported as the most-used solution, followed by H_2_O_2_, HNO_3,_ and enzyme digestion for MP detection in BMs. About the five studies in which *D. trunculus* was analyzed, only two used the 10% KOH protocol [25,34]. Then, a flotation step with NaCl was also carried out. Accordingly, the saturated NaCl solution was the most used for the flotation step in previous studies [25], due to its low cost and low chemical risk [60]. In addition, it has also been proven efficient for the detection of low-density polymers, such as polystyrene (PS), polyethylene (PE), polypropylene (PP), polyamide (PA), ethylene vinyl acetate (EVA), and polymethyl methacrylate (PMMA) [61,62]. These represent the main polymers found in the environment including marine sediments [63]. However, denser polymers such as polyethylene terephthalate (PET) and polyvinylchloride (PVC) could be extracted due to the change induced by weathering or other processes [64]. Even though the flotation step is not a must when using 10% KOH, this method allowed for the best separation of MPs [65]. It is reported that the MPs recovery rate in protocols involving a basic digestion with 10% KOH combined with a NaCl flotation reaches 90–95% [63]. Finally, 5 µm filters were used because they are the most widely used in the literature for MP detection in bivalves, even if very different pore sizes, ranging from 0.2 µm to 80 µm, have been used [25,66].

#### 3.3.2. Item Abundance

Items found in the procedural blanks and in the blank filters were morphologically analyzed and compared with those found in the samples. In this way, the items of similar appearances were eliminated from the total counting. Out of the 39 samples, 35 (89.74%) were positive, with a total of 85 items found. Moreover, in 53.85% (*n* = 21) of the samples, one or two items were found, while, in 35.90% (*n* = 14), more than two items were found (maximum eight items). In the other samples (10.26%; *n* = 4), no items were found. Overall, the MA of the items was 0.23 ± 0.17 items/g ww with a maximum number of 0.8 items/g ww, and 2.18 ± 1.78 items/35 individuals (0.06 ± 0.05 items/individual) (Table 3). In the literature, most studies reported an abundance of items/g or of items/individual. Abundance expressed as items/g is usually reported as wet weight (ww) (as in this study), although in a minority of cases, it has also been reported as dry weight (dw) [67]. The lack of standardization in reporting MP abundance values is an issue also highlighted by the EFSA (2016) [13,25,53]. Due to this, a comparison between the results obtained from different studies should be made carefully, and the use of different analytical methods, the units of measurement, and the number of samples should be especially considered. In studies in which *D. trunculus* specimens were analyzed, the relative MAs are reported as items/individual (60%; n = 3), items/g ww (20%, *n* = 1), and in both ways (20%; *n* = 1) (Table 4). In the study of Ben-Haddad et al. [32], the MA, expressed in items/g ww, is higher than that found in our study. However, the analyzed specimens of *D. trunculus* were not harvested in the Mediterranean Sea, but rather along the Atlantic coast of Morocco, so that the level of MP contamination is not the same. Also, both the MA values found in the studies conducted in the Black Sea are higher than this study, in terms of items/individuals (Table 2). Interestingly, our MA falls within the reported MA range of the unique study conducted in the Tyrrhenian Sea [33] (Table 2). Instead, in the other study conducted in Mediterranean Sea (Catalan coast), the MA values found were higher [36] (Table 2). Interestingly, in the same study, no significant difference between 48 h purified and non-purified *D. trunculus* samples were found [36]. Higher MAs were observed in another co-generic species (*D. cutaneus*) [68], and, more generally, in the entire Donax genus [69]. Of course, even in these cases, high MA values could be related to the collection site.

The literature considered for the comparison [25,53] reported the MP abundances according to four BM categories, namely clams, mussels, oysters, and scallops. To note, in both the reviews, the MA was reported as MPs/g ww, confirming how this unit is most suitable for comparing MAs. In particular, the calculated MAs are reported in Table 3.

Although the MAs reported by Danopoulus et al. [53] were lower overall in all the categories, with respect to Ding et al. [25], clams were found as the category with the highest MA in both the reviews (Table 3). In detail, Danopoulus et al. [53] reported 1.25 ± 0.54 MPs/g ww, and Ding et al. [25] reported 3.2 ± 4.2 MPs/g ww.

The higher MA of MPs in clams could be related to the fact that they are classified as infaunal bivalves living within the substrate, whereas mussels, oysters, and scallops are all epifaunal bivalves living above the substrate. Therefore, the differences in item numbers could be attributed to the different MPs present in the two habitats (water and substrate, respectively) [70,71]. *D. trunculus* belongs to the order of Veneroida as clams, and lives on the sandy seabed, being exposed not only to the suspended MP water column, but also to the plastic material that is deposited. However, in this study, a lower MA with respect to clams was found. Interestingly, in the study of Exposito et al. [36], in which six species of BMs among infaunal (*D. trunculus*, *Ensis siliqua*, *Ruditapes decussatus*) and epifaunal *(Mytilus galloprovincialis*, *Crassostrea gigas*; *Bolinus brandaris*) bivalves were analyzed, *D. trunculus* presented significantly lower levels of items per individual with respect to the other species analyzed; this is likely due to its low ability to ingest large MPs. Moreover, in the same study, clams (*R. decussatus*) and mussels (*M. galloprovincialis*) showed higher MAs in terms of MPs/g ww. Also, in another study conducted on specimens of these two species collected in the bay of Izmire (Turkey), similar MA values, in terms of MPs/individual, were found [72]. Regarding clams, these may accumulate microfibers and microfragments which may be more difficult to expel, and, because they are infaunal, they are also affected by sediment MP contaminant [70,71,73,74]. On the contrary, mussels can accumulate more MPs due to their size, high pumping, and filtration rates [58,70,75]. Thus, the MPs’ filtering efficiency and accumulation probably depends on size, filtering selection, and environmental conditions [36]. Due to these differences, more than one species, selected among epifaunal and infaunal, should be analyzed to produce a better monitoring of MPs [25].

#### 3.3.3. Positive Samples per Collection Point and per Season

In this study, the low total number of collected samples, associated with the differences in their number per collection point and season, allowed for the calculation of the percentage of positive samples per site and per season. Therefore, we provide only a comparison of the positivity rates to eliminate differences due to the different sample numbers per collection point and per season.

With respect to the collection points, 100% of the samples collected in VL were found to be positive regarding items, followed by those of Gombo SGT and Gombo Pi (88.88%), CCT (87.50%), and VP (83.33%) (Table 3). Interestingly, VL was the only site where more than two items were found in most samples (71.00%).

With respect to the season, 100% (*n* = 8) of the samples were found to be positive in spring, followed by summer, autumn, and winter, in which 88.24%, 85.71%, and 85.71% of the samples, respectively, were positive (Table 3).

With respect to the season trend in BMs, the review by Ding et al. [25] reported higher MAs in autumn, while the MAs in spring/summer only ranked third and second, respectively, among season [25]. Contrariwise, the lower MA observed in winter is in line with our findings. The MA seasonal trend observed in the literature for *D. trunculus* are different, according to the analyzed study [32,35]. For instance, the highest MA of items in specimens collected during the summer season is explained by some authors in relation to the greater MP accumulation, which was caused by the increase in tourist activities, fishing, and industrial processes, and also by the intensification of the metabolic and reproductive activities of *D. trunculus* during this period [33,35,76]. Previous studies found that BMs collected from areas with intensive human activities contained a higher number of items [77] and highlighted a correlation between the abundance of items in BMs and the surrounding environment [78,79]. Therefore, the highest positivity found in the spring/summer season in our study can be linked to the increase of the anthropic presence, related to tourist activities in this part of the Tuscan coast. This thesis could be supported by the fact that most of the items chemically identified in this study were PET polyester fibers that are widely used in textile industries (see Section 3.5). Moreover, during this period, items are more available for these BMs as they tend to deposit, whereas, in autumn and winter, waves and winds can cause the water to stir, thus causing items to migrate [80].

#### 3.3.4. Items Shape, Size, and Color

**Figure 4:**
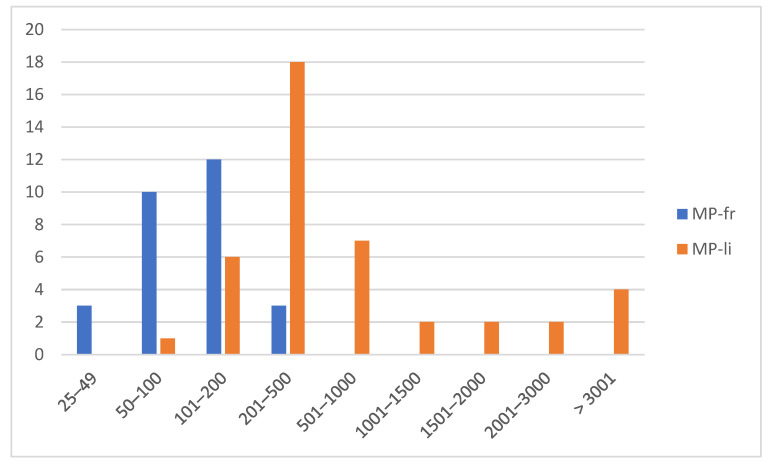
Distribution of items in relation to size and shape (MP-fr; MP-li).

Based on GESAMP (2019) [14], 45 items were classified as MP-li (52.94%), and 40(47.06%) were identified as MP-fr (Figure 4). The items were of various colors, including black (29%) and blue (28%), followed by white (16%), brown (8%), grey (7%), purple (5%), green (4%), and red (1%) (Figure 3).

In particular, the most frequently encountered color for MP-fr was black (43%), while for MP-li, it was blue (40%) (Figure 4). This result is in line with previous studies conducted in the same species (see Table 3). The percentage of fibers (MP-li) was also statistically higher in most of the studies selected in the review by Ding et al. [25], analyzing different BM species. Fibers represent the most-found MP type in seawater, freshwater, and sediment samples [81,82,83,84]. Anthropogenic fibers are mainly derived from textile industries and can be divided into three categories: natural fibers, such as cotton and wool; semi-synthetic fibers, such as rayon, which are reconstituted from the dissolved cellulose of plant materials and shaped into fibers via extrusion; and synthetic fibers from petrochemical-based compounds [85]. Nevertheless, the principals’ sources of fibrous material in the marine ecosystem might be untreated sewage and debris, such as torn fishing nets and broken ropes [86,87,88]. Fiber predominated in BMs collected from China, Thailand, and many European countries, while fragments are more reported in bivalves from Vietnam, France, Greece, and Brazil, reflecting the different types of plastics used [89]. Our results are also in line with those reported in the review of Santini et al. [90], in which microfibers released from synthetic fabrics represent about 40% (1.6–84.9%) of the MPs in the water column and sediments of the Mediterranean Sea, followed by fragments (34.5%; 1.6–72.7%). However, it must be considered that filaments tend to be overestimated, being more easily detected than other types of items [2,49]. In fact, Song et al. [50] reported that using the stereomicroscope can determine an overestimation of fibers and an underestimation of fragments. On the contrary, using the Fourier transformed infrared (FTIR) spectroscopy, more fragments are detected. Concerning the colors, dark items (black, blue, green, red) are more ingested by BMs. In fact, dark items may attract predators and increase the likelihood of ingestion, due to prey item resemblance [91]. In addition, a possible underestimation of transparent items could happens considering that colored items are more easily visible. However, light items (pink, transparent) were also found in the soft tissue of many BM species [32,89,91,92]. In fact, in some studies, non-colored and light-colored MPs (transparent, white, light blue), that could be the result of an alteration or loss of their original colors caused by environmental weathering processes, were the most common, followed by black and blue [66,89,90,93]. Finally, it must be considered that, although measures to avoid contamination were adopted in this study, it is possible that some fibers may be of environmental origin.

Using the ImageJ software version 1.52t, 70 out of the 85 items (82.35%) were measured. Of these, 28 were classified as MPs-fr (40%) and 42 as MPs-li (60%). Items with sizes between ~201 µm and ~500 µm (*n* = 21; 30%) were the most found, followed by those with sizes between ~101 µm and ~200 µm (*n* = 18; 25.71%), and those with size between ~50 µm and ~100 µm µm (*n* = 11; 15.71%) (Figure 3). Specifically, MPs-li showed sizes between ~100 µm and ~4000 µm in length, but about half (*n* = 18; 42.85%) were in the ~201 µm to ~500 µm range. The length of the MPs-fr ranged between ~30 µm and ~500 µm, with a predominance of those between ~101 µm and ~200 µm (Figure 4).

The item sizes were like those found in the studies reported in Table 3, ranging from ~25 to ~2000 µm. Accordingly, also in the studies selected in the review by Ding et al. 2022, the percentage of items < 1000 µm was higher than those between 1000 and 5000 µm. This suggests that the smaller items are more easily ingested by BMs, being like its natural food items [68,94]. In this regard, it is known that particles > 100 µm are unlikely to be ingested by BMs due to the anatomical constraints of the gills and mouth. Particles of this size, corresponding to the most part of those detected in the present study, once captured by the gills, are usually transported towards the mantle and rejected as pseudofeces. This process reduces the risk of adverse effects for BMs, due to mechanical blockages of the digestive tract and the absorption of contaminants released by the particles [23]. However, in some areas, including Italy, South Africa, and China, MPs between 1000 and 5000 µm were the most abundant in mussels and clams [47,87,95]. Nevertheless, the wide size range could also be related to the pore size of the filters used for item extraction and the limitations of the technique used for polymer identification [53]. However, comparing data from the various studies is difficult, as there is no standard for selecting items and measuring particles [25].

### 3.4. Polymer Identification of Items

**Figure 5:**
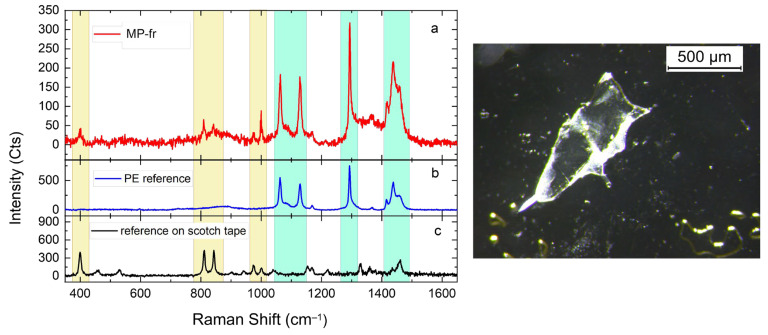
Comparison between the Raman spectra (**a**) of a micro fragment extracted from samples of *Donax trunculus,* analyzed for microplastics detection, the reference spectrum of PE (**b**), and the spectrum of the scotch tape (**c**) used during the analysis for transporting the extracted items.

Item identification via light microscopy serves as the first step in MP detection, but it is susceptible to human error [85] and requires analysis to reliably distinguish plastic materials from other compounds, such as natural fibers or organic material [90,96,97]. Spectroscopy techniques are effective in item polymer identification, but they are laborious, time-consuming, and expensive, making them difficult to use for routine investigations [98]. Accordingly, only three out of the five studies that investigated MPs in *D. trunculus* performed a chemical identification of the items (Table 2). Studies using spectroscopy for chemical identification usually test only a small percentage of particles [48,98], as in the present study. According to Ding et al. [25], the most frequently used methods for polymer identification were FT-IR and Raman spectroscopy. In this study, 18 items randomly selected from those extracted based on their morphological prevalence were analyzed by Raman spectroscopy. Out of the 18 items analyzed (21.17% of the total), 15 (83.33%) were confirmed to be MPs, and two were not made of plastic polymers. For the final one, it was not possible to make any identification due to the huge fluorescence background which prevented any Raman characterization, despite the excitation source being in the NIR spectral region (λ_exc = 785 nm), where the fluorescence emission is mostly absent or weak. The chemical identifications revealed that most of the tested items were made of PE followed by PET. Some items were instead identified as copolymers. Figure 5 shows an example of polymer identification. In detail, eight MP-lis and one MP-fr (red line in Figure 5) were identified as PE (60%). The reference spectrum acquired on scotch tape has a completely different signal, which matches with the Raman fingerprint of PP (Figure S4). Four (30%) MPs-frs were identified as PET, while one MP-li and one MP-fr were copolymers partially identified as Poly(styrene-ethylene-butylene) and Poly[(2,3-epoxypropyl methacrylate):Styrene:Ethylene Dimethacrylate)], respectively (Figure S2). The two MPs-li non-plastic items were identified as cotton and microcrystalline cellulose (Figure S3).

On the one hand, the chemical identification of items allows for assessment of the real level of MP contamination; on the other hand, it provides useful information for tracing MPs’ sources [99]. From a risk assessment perspective, polymer identification allows the evaluation of possible overestimates in the number of MPs identified by using the stereomicroscope [48]. In this regard, in the present study, two MPs-lis, considered as potential MPs under the stereomicroscope, were later identified as cotton and cellulose. The occurrence of cotton fiber could be indicative of airborne contamination, a recurring phenomenon in the microplastic study [100], while the cellulose fiber could be algae captured by the *D. trunculus* from the marine environment.

PE, the polymer most detected in this study, was also reported as the predominant polymer in the study of Ben-Haddad et al. [32], while in Olivieri et al. [33], a predominance of PET, polyvinylidene chloride (PVDC), and nylon was reported. In Exposito et al. [36], a predominance of polyester (PES), polyvinylidene fluoride (PVDF), and then PE was reported (Table 2). The other two studies that analyzed Trucate donax did not perform any chemical identification of items (Table 2). PE was also the prevalent polymer found in another species belonging to the Donax genus (*D. cutaneus*) collected in India, while in a study conducted on the Donax genus (China), the prevalent polymers were cellophane (CP) and PET [68,69]. Additionally, in another study conducted in South Korea, PE was also found among the prevailing polymers of clam species belonging to a different genus [89]. In the review conducted by Ding et al. [25], PET was the most frequently reported polymer, followed by PE, rayon, PP, cellophane, PES, PA, PVC, PS, and acrylic [25]. These authors, therefore, reported PE and PET polymers to be the two main contributors to global MP pollution in BMs. Their reviews showed that PET was the dominant polymer in BMs from China, South Africa, Mexico, and Portugal, whereas PE dominated a large proportion of MP composition in bivalves from Greece, France, New Zealand, and Italy (according with our study) [71]. An earlier review, conducted by Cavalca Bom and Sà [101], also reported PE and PET as prevalent polymers in MBs, also showing a correlation between these polymers and those found in the marine environment [102]. Indeed, polymers such as PE, PP, PET, PVC, and PA, used for packaging materials, are the common sources of plastic pollution [62,101]. Moreover, polymers such as PE, PP, PET, and PA are largely used to produce fishery tools like ropes, nets, buoys, and tubes [103]; this could produce a large amount of MPs because of long-term exposure to sunlight and mechanical damage [49]. Additionally, MPs in PETs discharged into the marine environment with sewage can originate from the breakdown of clothes and carpets. Indeed, PET polyester fibers have been widely used in textile industries since the 1990s [104,105]. All these findings are in line with the global data on plastic production, with PE representing 26.9%, and PET representing 6.2% [106]. However, it must be emphasized that the differences in polymer MAs observed between BM species also depend on their natural habitat (and thus on the density of the particles), their method of production, and their feeding characteristics [36]. Finally, regarding the styrene-ethylene-butylene copolymer identified in the present study, this is used in road materials to make the asphalt more elastic [107].

### 3.5. MP Abundance and Human Exposure

The MA calculated based on the item numbers was then recalculated after the polymer identification (corrected MA). In fact, considering that only 83.33% of the items were identified as MPs, the correct MA would be 0.18 MPs/g ww. Our results regarding the MPs could be considered as representative of the current MP contamination in the investigated area. In fact, samples derived from five harvesting areas (G-SGT, G-PI, CCT, VL, VP) all classified as Class A in 2021, according to Reg. (UE) 625/17 [39]. BMs harvested in these areas are therefore directly intended for human consumption without having to undergo prior depuration treatment (as in the case of BMs harvested in Class B or Class C areas) [39]. Several studies reported that the depuration process, which BMs undergo, is an important variable in defining the MA [5,108]. However, no significant differences in MAs between depurated and non-depurated BMs were found, although this could be due to insufficient time to remove the MPs from organisms [36].

Based on the corrected MA, *D. trunculus* consumers could be exposed to 19.2 MPs/per capita/year, according to the annual consumption of clams (106.6 g). This value is lower than that provided by Ben-Haddad et al. [32] (1072 MPs/capita/year ranging from 560 to 1897.6). On the contrary, in the study of Exposito et al. [36], they estimate a mean annual consumption of MPs per capita for the adult population of Catalonia to be 8103 (MPs/per capita/year), suggesting how BMs consumption could be a crucial cause of exposure [36]. In a study on risk assessment related to the consumption of BMs at a global level, an intake of 715 (ranging from 15 to 7333) MPs/capita/year was estimated on the basis of the global average consumption of molluscs of 367 g of pulp (considering the data from FAOSTAT and the ratio (value: 3) of the whole body weight–the soft tissue weight, as reported by Cho et al. [89] [73,89]). Nevertheless, the human exposure to MPs is very different between countries due to geographical and cultural differences in BM consumption [73]. Considering the global mean consumption established in the study of Ding et al. [73] (367 g of soft tissue/capita/year), the MP intake found in our study would still be much lower (66.1 MPs/capita/year). A high risk of exposure, calculated based on annual BM consumption and the MAs of MPs per gram, was found in countries such as China and South Korea, while at the European level, high risks was found in France and Greece [49].

Although we did not identify all items (only the 21.17%), we also performed the MP risk assessment on human health using the model proposed by Lithner et al. [109]. In particular, the risk was calculated based on Xu et al. [104], who considers the percentage of MP polymer types detected in *D. trunculus* and their relative hazard scores [109]. Then, the health risk level of MPs based on the polymer risk index was evaluated according to the hazard grades (1 to 10,000), which was classified into five levels of hazards (I, II, III, IV, and V) [109,110,111]. According to the percentage of PE MPs (60%) and PET MPs (30%) found in this study (not considering the remaining two copolymers), the health risk level of MPs was classified as level III (moderate). Interestingly, Italy and other countries (China, South Africa, New Zealand, South Korea, and the USA) were also classified at this level of risk in the study of Ding et al. [73]. In the latter study, countries classified at level IV (high level risk) presented a high percentage of polymers with high hazard scores, such as PCV (Sn = 10,551), while polymers like PE (Sn = 11) and PET (Sn = 4) presented a lower hazard score.

## 4. Conclusions

In this study, *D. trunculus* specimens collected along Tuscan coasts under official monitoring, conducted by the LHA of the Tuscany region, were analyzed with respect to the occurrence of MPs. A low MA of MPs was found when compared to the other studies available for the same species in other areas. This study also found that polymers identified in the analyzed specimens can be classified as moderate risks. Despite the limitations of the study in terms of chemically analyzed items, the outcomes from this study can implement the literature with new data, ensuring reliability guaranteed by the use of strict quality control measures. In particular, this study provides new data on a less-investigated species collected from an area never investigated.

## Figures and Tables

**Table 1:** Studies available in the literature, investigating MP occurrence in *D. trunculus*.

Date of Collection	Location	N° of Collection Point	N° or Tissue (g) per Collection	Total N° or Tissue (g) Collected	Total N° or Tissue (g) Analyzed	Reference
2020 June	Black Sea (Turkey)	2	-	51	51	Senturk et al. [34]
2022 April–May June–July	Black Sea (Bulgaria)	9	500 g	4500 g	90	Alexandrova et al. [35]
2018–2019 (Summer and winter seasons)	Atlantic Ocean (Morocco)	6	120	720	720	Ben- Haddad et al. [32]
2017 October–November	Mediterranean Sea (Catal Coast, Spain)	8	2000–4000 g	16,000–32,000 g	1632/424.2 g	Exposito et al. [36]
September 2020– June 2021	Mediterranean Sea Mediterranean Sea (Tyrrhenian Sea, Latium Coast, Italy)	1	15	150	50–150	Olivieri et al. [33]

**Table 2:** The abundance of MPS (range), dominant shape, color, size, and polymer of MPs detected in *D. trunculus*. PE = polyethylene; PP = polypropylene; PES = Polyester; PVDF = polyvinylidene fluoride; PET = polyethylene terephthalate; PVDC = polyvinylidene chloride; FT-IR: Fourier Transform Infrared Spectroscopy analysis; ATR: Attenuated Total Reflectance.

Abundance of MPs/Items (Range)	Dominant Shape	Dominant Color	Dominant Size (µm)	Dominant Polymer	Pylimer Identification Method Used	Percentage of Items Identified	Reference
1.65 MPs/individual	Fiber Films Fragment	Blue Black, blue Green	1000–2000 100–500	-	-	-	Senturk et al. [34]
2.52 ± 0.72 (0.31–4.46) MPs/individual	Pellet Fiber Fragment	NR	<25	-	-	-	Alexandrova et al. [35]
3.35 ± 1.58 (1.75–5.93) MPs/g ww	Fiber Fragment	Blue, yellow Blue, green	100–500 500–1000 <100	PE (65%) PP (35%)	ATR-FTIR and FTIR	100%	Ben-Haddad et al. [32]
1.04 ± 0.89–2.56 ± 1.18 MPs/g ww 0.32 ± 0.27–0.67 ± 0.31 MPs/individual	Fiber Fragment films	NR	20–1000	PES PVDF PE	ATR-FTIR and FTIR	100%	Exposito et al. [36]
0–0.56 MPs/individual	Fiber	Black Blue	100–1000	PET (60%) PVDC (20%) Nylon (20%)	FTIR	100%	Olivieri et al. [33]
0.23 ± 0.17 items/g ww 0.06 ± 0.05 items/individual 0.18 MPs/g ww (after the identification)	Fiber Fragment	Black Blue White	201–500 101–200 20–100	PE (60%) PET (30%)	Raman	21.2%	This study

**Table 3:** MA values for the four BM (bivalve mollusc) categories (clam, mussel, oyster, and scallop), reported in the two reviews used for the comparison.

BM Category	MA Value in Ding et al. [25] (MPs/g ww)	MA Value in Danopoulus et al. [53] (MPs/g ww)
Clam	3.2 ± 4.2	1.25 ± 0.54
Mussel	1.8 ± 1.4	0.57 ± 0.15
Oyster	1.3 ± 1.3	0.57 ± 0.36
Scallop	1.5 ± 1.6	0.48 ± 0.28

**Table 4:** Positive samples found according to collection point and season, with the relative percentage.

**Collection Points**	**Positive Sample**	**%**
CCT	7/8	87.50
Gombo PI	8/9	88.88
Gombo SGT	8/9	88.88
VL	7/7	100.0
VP	5/6	83.33
**Seasons**	**Positive Sample**	**%**
Spring	8/8	100
Summer	15/17	88.23
Autumn	6/7	85.71
Winter	6/7	85.71

## Data Availability

Data are included in the manuscript and available in Supplementary Materials attached to the text; further data are also available from the authors upon request.

## Supplementary Materials

The following supporting information can be downloaded at: https://www.mdpi.com/article/10.3390/ani14040618/s1, Table S1: Specimens collected within the five production areas with code, collection date, and collection site (VL: Viareggio Levante; VP: Viareggio Ponente; G-Pi: Gombo Pisa; G-SGT: Gombo San Giuliano Terme; CCT: Centro Coni and Tirrenia); Figure S1: Raman spectrum of a cellulose microfiber (red line) extracted from samples of *Donax trunculus*. At the time of Raman analysis, microfiber was placed onto scotch tape previously attached to a glass slide and covered with a glass coverslip. The black line represents the spectrum of the background collected before the microfiber analysis, and the reference of the glass can be observed at the same height; Figure S2: Raman spectra of a microfiber (a) and a microfragment (b) and the related reference library spectra, extracted from samples of *Donax trunculus*. Both microitems were identified as plastic copolymers; in detail, microfibers as Poly(styrene-ethylene-butylene) and microfragments as Poly[(2,3-epoxypropyl methacrylate):Styrene:Ethylene Dimethacrylate)]; Figure S3: Raman spectra of a microfiber (blue line) and the related reference library spectrum (red line) extracted from samples of *Donax trunculus*. The microfiber was identified as microcrystalline cellulose; Figure S4: Raman spectrum of scotch tape (black line) used to transport microitems extracted from samples of *Donax trunculus*. The spectrum matched with the Raman fingerprint of polypropylene (red line).
