# Supplementary material for: The Occurrence of Microplastics in Donax trunculus (Mollusca: Bivalvia) Collected along the Tuscany Coast (Mediterranean Sea)
Source: Animals (Basel). 2024 Feb 14;14(4):618. doi: 10.3390/ani14040618 (PMC10886031; doi:10.3390/ani14040618)
Supplement: Supplementary file 1 [file animals-14-00618-s001.zip › animals-2811460-supplementary.pdf]

Table S1. Specimens collected within the five production areas with code, collection date and collection site (VL: Viareggio Levante; VP: Viareggio Ponente; G- Pi: Gombo Pisa; G-SGT: Gombo San Giuliano Terme; CCT: Centro Coni and Tirrenia).

Sample code	Collection date	Collection site
1T	16/2/2021	VL
2T	16/2/2021	VP
3T	3/3/2021	GOMBO Pi
4T	3/3/2021	CCT
5T	11/3/2021	VP
6T	11/3/2021	VL
7T	23/3/2021	G- SGT
8T	23/3/2021	G- PI
9T	31/5/2021	CCT
10T	31/5/2021	G- SGT
11T	1/6/2021	VP
12T	1/6/2021	VL
13T	11/6/2021	G- Pi
14T	11/6/2021	G- SGT
15T	23/6/2021	G- SGT
16T	23/6/2021	CCT
17T	30/6/2021	G- Pi
18T	30/6/2021	VL
19T	30/6/2021	VP
20T	22/7/2021	G- SGT

21T	22/7/2021	G- Pi
22T	22/7/2021	CCT
23T	22/7/2021	VL
24T	22/7/2021	VP
25T	13/8/2021	CCT
26T	13/8/2021	G- Pi
27T	26/8/2021	VL
28T	26/8/2021	G- SGT
29T	26/8/2021	CCT
30T	16/9/2021	G- Pi
31T	16/9/2021	G- SGT
32T	13/10/2021	G- SGT
33T	13/10/2021	CCT
34T	27/10/2021	G- Pi
35T	15/12/2021	CCT
36T	18/11/2021	VL
37T	18/11/2021	VL
38T	24/11/2021	G- Pi
39T	24/11/2021	G SGT

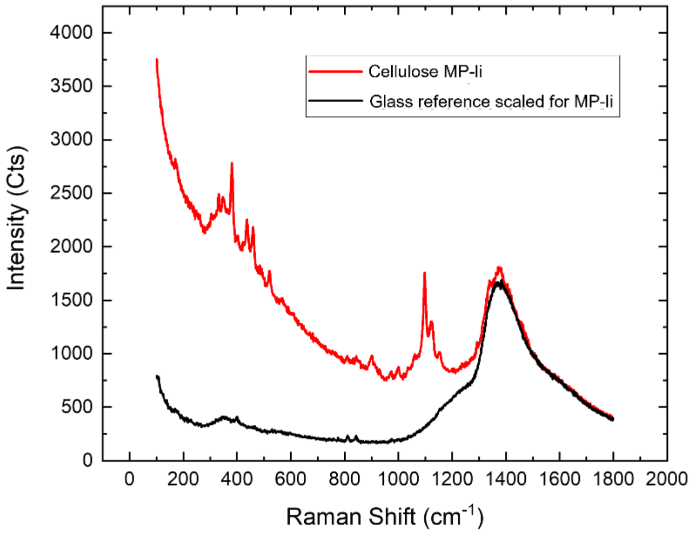

Figure S1. Raman spectrum of a cellulose microfiber (red line) extracted from samples of *Donax trunculus*. At the time of Raman analysis, microfiber was placed onto scotch tape previously attached to glass slide and covered with a glass coverslip. The black line rapresents the spectrum of the background collected before the microfiber analysis and the reference of the glass can be observed at the same height.

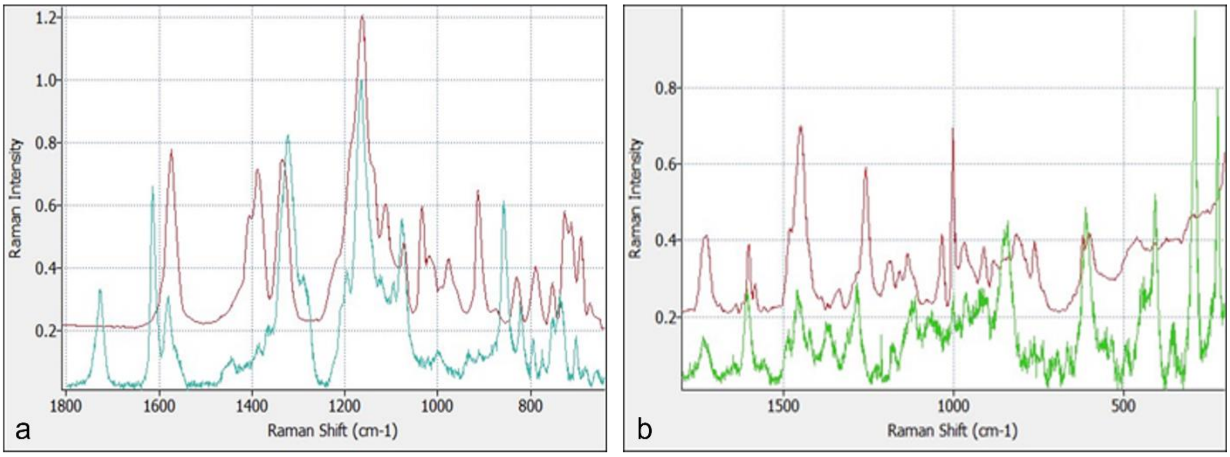

Figure S2. Raman spectra of a microfiber (a) and a microfragment (b) and the related reference library spectra extracted from samples of *Donax trunculus*. Both microitems were identified as plastic copolymers; in detail, microfiber as Poly(styrene-ethylene-butylene) and microfragment as Poly[(2,3-epoxypropyl methacrylate):Styrene:Ethylene Dimethacrylate)].

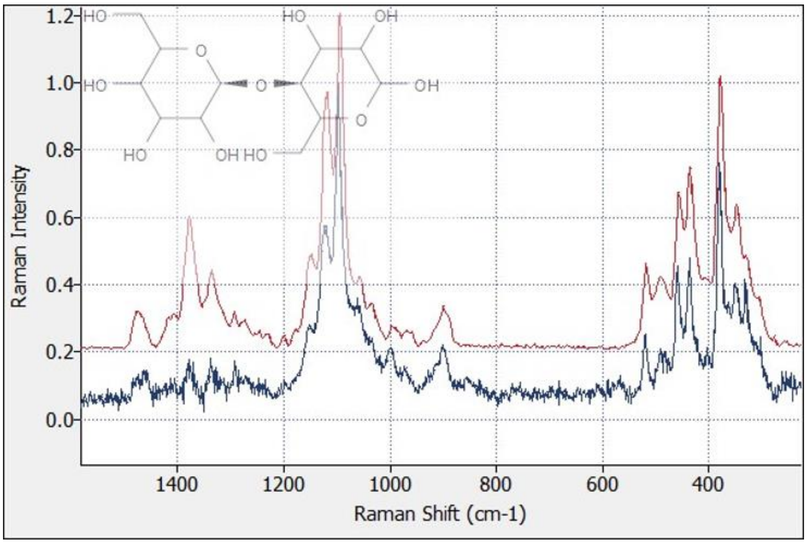

Figure S3. Raman spectra of a microfiber (blue line) and the related reference library spectrum (red line) extracted from samples of *Donax trunculus*. The microfiber was identified as microcrystalline cellulose.

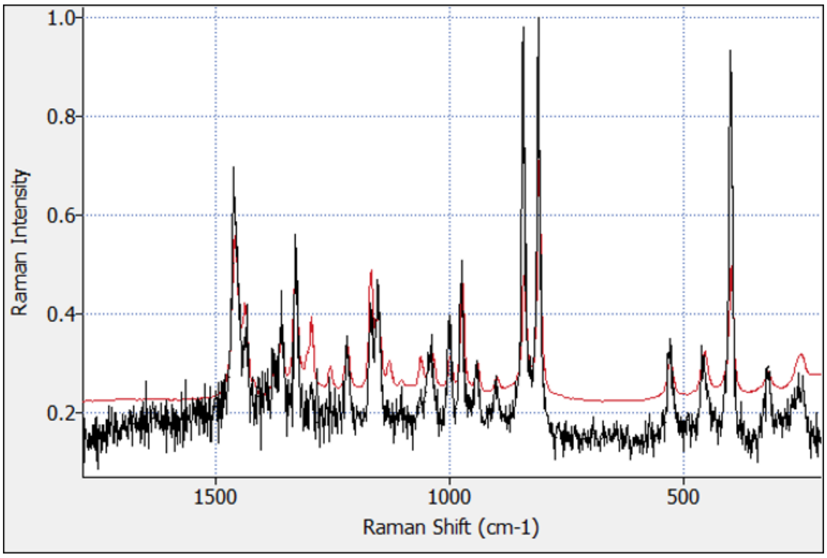

Figure S4. Raman spectrum of scotch tape (black line) used to transport microitems extracted from samples of *Donax trunculus*. The specrtum matched with the Raman fingerprint of polypropylene (red line).
